# Fu Brick Tea Protects the Intestinal Barrier and Ameliorates Colitis in Mice by Regulating Gut Microbiota

**DOI:** 10.3390/foods14071122

**Published:** 2025-03-24

**Authors:** Yangbo Zhang, Haiyan Lin, Yifan Xiong, Zhixu Zhang, Li Zeng, Zhonghua Liu

**Affiliations:** 1 School of Pharmacy, Shaoyang University, Shaoyang 422000, China; zhangyblucky@163.com; 2 Key Laboratory of Tea Science of Ministry of Education, Hunan Agricultural University, Changsha 410128, China; linhaiyan1003@163.com (H.L.); 18229966792@163.com (Y.X.); 3 Yuelushan Laboratory, Changsha 410128, China

**Keywords:** ulcerative colitis, Fu Brick tea, ZO-1, gut microbiota, *Akkermansia*

## Abstract

Ulcerative colitis (UC) pathogenesis is strongly linked to gut microbiota dysbiosis and compromised intestinal barrier integrity. Emerging evidence suggests that targeted dietary interventions may restore microbial homeostasis and ameliorate colitis progression. In this study, we evaluated the therapeutic potential of Fu Brick tea (FBT) using a dextran sulfate sodium (DSS)-induced murine colitis model. The results indicated that oral administration of FBT extract significantly improved the disease index, reduced inflammatory response, protected intestinal barrier protein (e.g., ZO-1), and maintained intestinal structure integrity. Furthermore, FBT intake increased the diversity of gut microbiota, promoted the growth of beneficial bacteria (e.g., *Akkermansia*), inhibited the proliferation of harmful bacteria (e.g., *Desulfovibrioceae*, *Escherichia*, and *Helicobacter*), restored intestinal homeostasis, and alleviated colitis symptoms including diarrhea. These findings position FBT as a promising nutraceutical candidate for UC management via multi-target modulation of mucosal immunity and microbial ecology.

## 1. Introduction

Inflammatory bowel disease (IBD) is a chronic inflammatory disorder of the gastrointestinal tract, predominantly involving colonic mucosa [1]. It comprises two clinical subtypes: Ulcerative colitis (UC) and Crohn’s disease (CD). Characteristic pathophysiological manifestations include chronic diarrhea, hematochezia, weight loss, persistent mucosal inflammation, and ulcerative colonic lesions [2]. With a global prevalence exceeding 6.8 million cases and rising incidence rates in China, IBD imposes significant socioeconomic and healthcare burdens [3]. Although its precise etiology remains elusive, emerging evidence implicates gut microbiota dysbiosis as a pivotal contributor to IBD pathogenesis. Comparative analyses reveal diminished microbial diversity and altered bacterial composition in IBD patients relative to healthy controls [4]. This ecological imbalance, coupled with dysregulated inflammatory signaling, drives disease progression through multiple mechanisms.

Microbial metabolites modulate host immunity by initiating non-specific immune responses that stimulate inflammatory mediator production and reactive oxygen species (ROS) generation [5,6]. These processes exacerbate epithelial barrier dysfunction, particularly affecting intestinal crypt architecture and goblet cell homeostasis [7,8]. Current therapeutic strategies primarily employ biologics, including vedolizumab, TNF-α antagonists, and ustekinumab [9]. However, their high costs and adverse effect profiles necessitate the development of safer, cost-effective alternatives for UC management.

The gut microbiota constitutes a dynamic ecosystem of trillions of microorganisms that critically influence host physiology through diet-modulated interactions [10]. Intestinal mucus layers and epithelial barriers collectively prevent the translocation of luminal antigens. Dysbiosis-induced mucosal inflammation arises from synergistic interactions between microbial ecology disruption and inflammatory factor dysregulation. Gut microorganisms fundamentally regulate multiple host functions, including immune responses, nutrient metabolism, and maintenance of intestinal homeostasis [11].

Microbial translocation into the lamina propria triggers pathological immune activation, amplifying inflammatory mediator release and ROS production [12,13]. This cascade perpetuates epithelial barrier damage through structural and functional impairment of specialized intestinal cells. Experimental evidence demonstrated that fecal microbiota transplantation could ameliorate intestinal motility and barrier integrity in murine colitis models [14], supporting the therapeutic potential of microbiota-targeted interventions [15,16]. Comprehensive characterization of microbial composition and function represents a critical step toward developing personalized microbial therapies for IBD.

Dark tea is one of the six major tea categories, and Fu Brick tea is one of the varieties of dark tea that has received the most research attention, attracting attention for its anti-inflammatory and antioxidant properties. Epidemiological studies have shown that drinking dark tea significantly reduces the risk of UC [17]. Wang et al. found that Fuzhuanbrick tea polysaccharide (FBTP) relieved the intestinal microbiota disorder induced by UC, contributing to the proliferation of beneficial microbiota, including *Latobacillus* and *Akkermansia*, followed by a significant increase in SCFAs levels. FBTP significantly increased the colonic expressions of aromatic hydrocarbon receptors (AhRs) and interleukin-22 (IL-22) and further promoted the expressions of intestinal tight junction (TJ) proteins ZO-1 and occludin in the colitis mice [18]. Relevant animal experiments have further confirmed the effective therapeutic effect of daily drinking dark tea on UC, and its side effects are relatively small [9,19]. However, although some studies have found that dark tea has a significant effect on alleviating DSS-induced colitis in mice, most studies have mainly focused on the regulatory effect of dark tea on inflammatory pathways and have not focused on its effects on the intestinal barrier and gut microbiota. Therefore, this study used a DSS-induced mice colitis model to explore multiple dimensions (including body weight, disease activity index, colon length, colon pathological morphology, serum inflammatory factors, intestinal barrier function, gut microbiota, etc.) to investigate the mechanism of FBT in improving DSS-induced colitis in mice and regulating gut microbiota. These findings will provide a new theoretical basis for the prevention and treatment of intestinal diseases and further support the potential of dark tea as a dietary supplement to protect intestinal health.

## 2. Materials and Methods

### 2.1. Test Materials and Equipment

Fu Brick tea (FBT) was obtained from the Hunan Yiyang Tea Factory. Dextran sodium sulfate (DSS) was purchased from Sigma-Aldrich (St. Louis, MI, USA). Enzyme-linked immunosorbent assay (ELISA) kits for IL-1β, IL-6, and TNF-α, as well as ZO-1 antibody, were sourced from Hunan AF Biotechnology Co., Ltd (Changsha, China). The ECL chemiluminescence substrate was procured from Bio-Rad Laboratories, (Hercules, CA, USA). The 16S rRNA sequencing was performed by Wuhan Metavi Company (Wuhan, China). The following equipment was used: a refrigerated centrifuge (MIKRO 22R, Hettich, Tuttlingen, Germany), a multifunctional microplate reader (Thermo Fisher Scientific, Waltham, MA, USA), and an upright fluorescence microscope (Nikon Corporation, Tokyo, Japan).

FBT Extraction Method: FBT was extracted using pure water at a ratio of 1:10 (*w*/*v*) by heating at 100 °C for 30 min. After filtration, the residue was subjected to a second extraction under the same conditions. The filtrates from both extractions were combined, freeze-dried, and stored at −80 °C until further use. The primary biochemical components of the FBT extraction were analyzed using high-performance liquid chromatography (HPLC) and the national standard method for biochemical component determination.

### 2.2. Animal Experiment Design

The animal experiment was approved by the Animal Medical Research Ethics Committee of Hunan Agricultural University (Approval No. 43322034). Fifty specific pathogen-free (SPF) grade, 8-week-old healthy male C57BL/6 mice were purchased from Hunan Slack Jingda Experimental Animal Co., Ltd (Changsha, China). (Production License No. SCXK (Hunan) 2019-0004). All C57BL/6 mice were raised in the SPF-level standard animal room of Hunan Agricultural University. The ambient temperature was maintained at 25 °C and a 12/12 h light–dark cycle. The mice had free access to water and food during the entire experiment.

**Figure 1:**
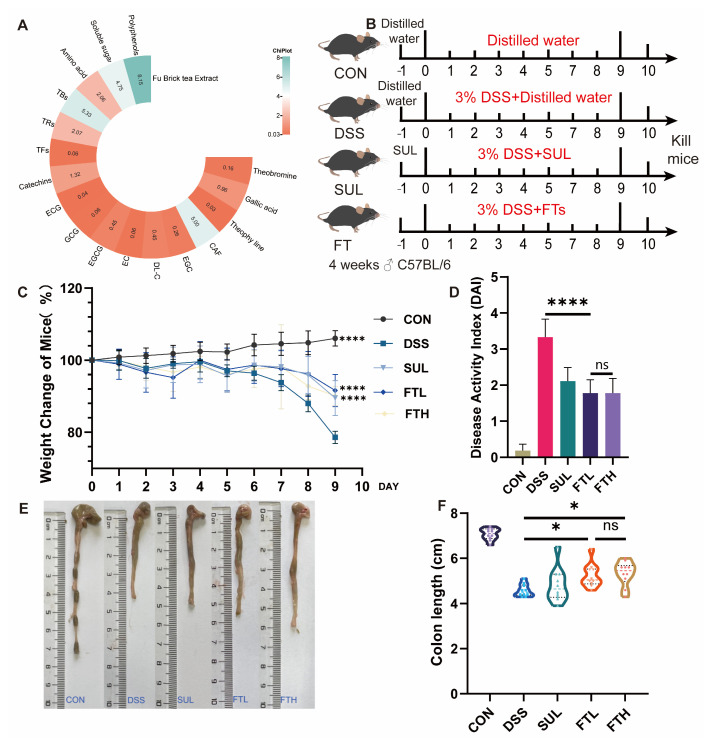
FBT alleviates DSS-induced colitis in mice. (**A**) Contents of main substances in FBT. (**B**) Experimental design. (**C**) Body weight changes of mice in each group. (**D**) Disease activity index of mice. (**E**,**F**) Effects of FBT on colon length of mice. *, *p* < 0.05; ****, *p* < 0.0001; ns indicates that the difference is not significant.

The experimental design is shown in Figure 1B. After 1 week of adaptive feeding, mice were randomly divided into the normal control group (CON), DSS enteritis model group (DSS), positive control group (sulfasalazine SUL), 200 mg/kg FBT group (FTL), and 400 mg/kg FBT group (FTH), with 10 mice in each group. The experimental period lasted 9 days. The drinking water of the mice in the CON group was distilled water. The drinking water of the mice in the DSS group, SUL, and FBT treatment groups for the first 7 days was distilled water with 3% DSS added to establish the mice colitis model. The mice in the FBT group were administered FBT water extract via gavage at doses of 200 mg/kg and 400 mg/kg, respectively, throughout the entire experimental period. The mice in the CON group and DSS group received the same volume of distilled water by gavage. The mice in the SUL group received the same volume of sulfasalazine by gavage. In addition, we monitored mice daily for symptoms of colitis, including body weight, fecal characteristics, and bleeding. After the experiment, the mice were anesthetized with 1% pentobarbital, and eyeball blood samples were collected. The contents of the colon and cecum were collected, and a part of the colon was cryopreserved at −80 °C for subsequent use. The other part of the colon was fixed in tissue fixative, and then tissue sections were performed to observe the colon morphology.

### 2.3. Disease Activity Index Evaluation

The physical condition of the test mice was checked and examined daily, and the consistency of the stool, whether there was bleeding, and the percentage of weight loss were recorded for a comprehensive assessment. The final disease activity index was calculated as follows: disease activity index = (stool consistency score + stool bleeding score + weight loss score)/3. The specific scoring criteria are shown in Table 1.

### 2.4. Detection of Inflammatory Factors in Serum

After allowing the mice blood to stand for 30 min, the samples were centrifuged at 4000 revolutions per minute (r/min) for 10 min at 4 °C to obtain the upper serum. Subsequently, the levels of inflammatory factors, including IL-1β, IL-6, and TNF-α, were assessed in the serum using the ELISA kit method.

### 2.5. Pathological Observation of Mice Colon Tissue Sections

The collected colon tissue was fixed in 4% polymethanol buffer for 24 h, then dehydrated and embedded in paraffin. Subsequently, the paraffin-embedded colon tissue was cut into sections with a thickness of 5 μm using a paraffin microtome, followed by the processing of these sections using hematoxylin and eosin (H&E) staining and periodic acid-Schiff (PAS) staining. The extent of colonic inflammatory infiltration, histopathological changes in crypt structure, ulceration and loss of crypts, presence or absence of ulcers, and presence of edema were then assessed, and these tissue samples were scored histologically.

### 2.6. Immunofluorescence Detection of ZO-1 Expression in Colon Tissue

After the colon paraffin sections were blocked with serum, the ZO-1 antibody was added and incubated at 4 °C overnight, and the secondary antibody was added dropwise and incubated for 1 h. The sections were then rinsed with PBS three times, DAPI was then used to counterstain cell nuclei, and finally, sections were sealed with Fluoromount-GTM. After sealing, pictures were taken with a fluorescence microscope, and the positive rate was calculated.

### 2.7. Gut Microbiota 16sRNA Detection and Analysis

The collected mice cecal contents were pretreated, genomic DNA was extracted using the CATB method, and then the extracted DNA was processed. The concentration and purity of DNA were determined with a NanoDrop 2000 UV–vis spectrophotometer (Thermo Scientific, Wilmington, NC, USA). The 16S rRNA sequencing and analysis were carried out according to Zhang et al. [20].

### 2.8. Detection and Analysis of SCFAs in Intestinal Contents

The SCFAs content was assessed by gas chromatography (GC, 6890 N, Antitron, Cleveland, OH, USA) with a flame ionization detector (FID) and HP-INNOWAX capillary column (30 m × 0.25 mm × 0.25 μm, Antitron). In short, the feces were dissolved in sterile water and centrifuged to remove the supernatant. The sample supernatant was mixed with an internal standard solution (0.3 mg/mL 2-ethylbutyric acid prepared in 0.2 mol/L HCl solution) to determine SCFAs. The conditions for GC analysis refer to Chen [21] et al.

The determination of acetate, propionate, and butyrate was performed on the Agilent 6890 N GC system equipped with an HP-INNOWAX column (30 m × 0.25 mm × 0.25 μm, Agilent Technologies, Santa Clara, CA, USA) and a flame ionization detector. At each run, 1.0 μL of pretreated sample was used with nitrogen as the carrier gas at a flow rate of 1.0 mL/min. Replenishment uses hydrogen, air, and nitrogen at flows of 30, 260, and 30 mL/min, respectively. Keep the oven temperature at 100 °C for 1 min, then heat up to 180 °C at 5 °C/min, and set the temperature of the sampler and detector to 250 °C for 4 min. The content of SCFAs in each sample was measured according to the abundance of the acetate, propionate, and butyrate standards.

### 2.9. Data Statistics

Sections and immunofluorescence images were obtained through CaseViewer software (3DHISTECH Ltd., version, 2.4.0., Budapest, Hungary); immunofluorescence protein analysis was completed using ImageJ software (version 1.8.0, National Institutes of Health (NIH), Bethesda, MD, USA), and the relevant positive expression area was calculated. The data were analyzed and processed using SPSS 28.0.0.0 statistical software, and graphics were drawn using GraphPad Prism 8.0.1 software. The results were expressed as mean ± standard deviation. One-way analysis of variance (ANOVA) and LSD test were used for analysis of variance. * *p* < 0.05 indicates that the difference is statistically significant. *, *p* < 0.05; **, *p* < 0.01; ***, *p* < 0.001; ****, *p* < 0.0001; ns indicates that the difference is not significant.

## 3. Results

### 3.1. Analysis of Functional Components of FBT

We examined the catechins, alkaloids, gallic acid, and other components in FBT, and the results are shown in Figure 1A. The content of catechins in FBT accounts for 1.32% of its dry weight, soluble sugar accounts for 4.75%, theaflavins are 0.06%, thearubigins are 2.07%, theabrownins are 5.33%, and the free amino acid content is 2.06%, and the total amount of polyphenols is 8.15%. Notably, after FBT has undergone fermentation and microbial treatment, the irritating substances in the tea have been oxidized and polymerized into other substances, resulting in a milder and less irritating taste of FBT.

### 3.2. Effect of FBT on Disease Activity Index of Colitis Mice

To investigate the alleviating effect of FBT on colitis, we administered FBT extract to mice while they drank 3% DSS (Figure 1B). The disease status of the mice in each group was observed throughout the experiment, and the colon length of the mice in each group was measured at the end of the experiment. As shown in Figure 1, compared with the CON group, after 9 days of ingestion of DSS, the mice showed a significant decrease in body weight (*p* < 0.005), symptoms of diarrhea, and bloody stools. However, after 9 consecutive days of oral administration of FBT extract, the mice showed a reduced trend of weight loss, and the symptoms of diarrhea and bloody stools were also alleviated.

As shown in Figure 1C, the disease activity index of the enteritis model group was significantly higher than that of the CON group, indicating that DSS caused colitis in mice, causing severe diarrhea, bloody stools, and weight loss. However, after the intervention of FBT, the disease index of mice with enteritis was reduced, and the disease status of the mice was improved. The colon length of mice in each group is shown in Figure 1E,F. It was measured that the colon length of the mice in the CON group was significantly longer than that of the mice in the DSS group, while the colon length of the mice in the FBT group was significantly longer than that of the DSS group, which suggests that FBT extract can prevent the shortening and fragility of the colon induced by DSS. In summary, diarrhea, bloody stools, and weight loss in colitis model mice were significantly improved after FBT intervention. In addition, FBT can also improve the symptoms of colon shortening in mice with DSS-induced colitis.

### 3.3. Effects of FBT on Inflammatory/Oxidative Factors of Colitis Mice

**Figure 2:**
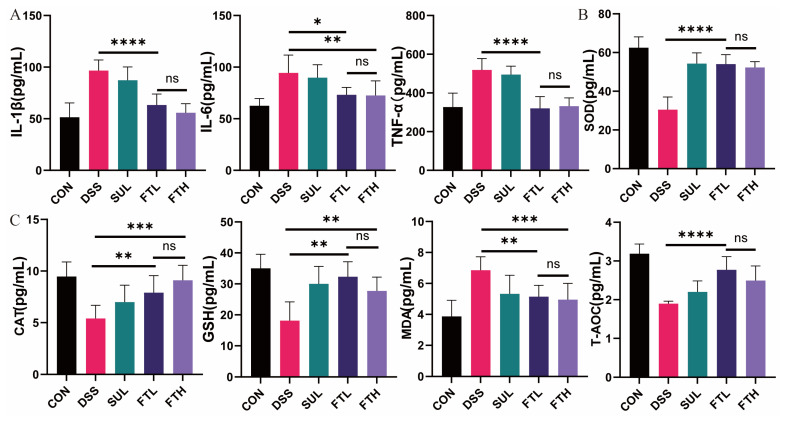
Effects of FBT on serum and intestinal antioxidant factors in mice with DSS-induced colitis. (**A**) Contents of inflammatory factors in mice serum. (**B**,**C**) Contents of oxidative stress-related indicators in mice intestines. *, *p* < 0.05; **, *p* < 0.01; ***, *p* < 0.001; ****, *p* < 0.0001; ns indicates that the difference is not significant.

To assess the effect of FBT on the inflammatory response in colitis mice, we used ELISA kits to detect the levels of inflammatory factors IL-1β, IL-6, and TNF-α in the serum. As shown in Figure 2A, the levels of IL-1β (*p* < 0.0001), IL-6 (*p* < 0.01), and TNF-α (*p* < 0.0001) in the serum of mice in the enteritis model group were significantly elevated compared with the CON group, indicating that DSS can trigger systemic inflammatory responses in mice. However, the levels of IL-1β (*p* < 0.0001), IL-6 (*p* < 0.01), and TNF-α (*p* < 0.001) were significantly reduced after oral administration of FBT compared with that of the DSS group, where the IL-1β and TNF-α content were restored to a level close to that of the CON group. This suggested that the intake of FBT can effectively reduce the inflammatory response induced by DSS in mice.

Inflammation in the body is often accompanied by intense oxidative stress. We detected MDA, SOD, CAT, GSH, T-AOC, and other oxidative stress-related indexes in the colons of mice (Figure 2B,C). The contents of antioxidant factors SOD, CAT, GSH, and T-AOC in the intestines of mice in the DSS group were significantly lower than those in the CON group, while the oxidative damage index MDA was significantly higher than that in the control group (Figure 2B,C). This indicated that DSS can trigger intense oxidative stress in the colon of mice. Compared with the DSS group, oral administration of FBT significantly increased and significantly decreased the contents of antioxidant factors SOD (*p* < 0.0001), CAT (*p* < 0.001), GSH (*p* < 0.01), T-AOC (*p* < 0.0001), and the level of MDA in the colon (*p* < 0.005). It is particularly noteworthy that oral administration of 400 mg/kg FBT extract had the most significant effect and can best alleviate the oxidative stress response of the colon.

### 3.4. Effects of FBT on Colon Morphology and Structure in Colitis Mice

**Figure 3:**
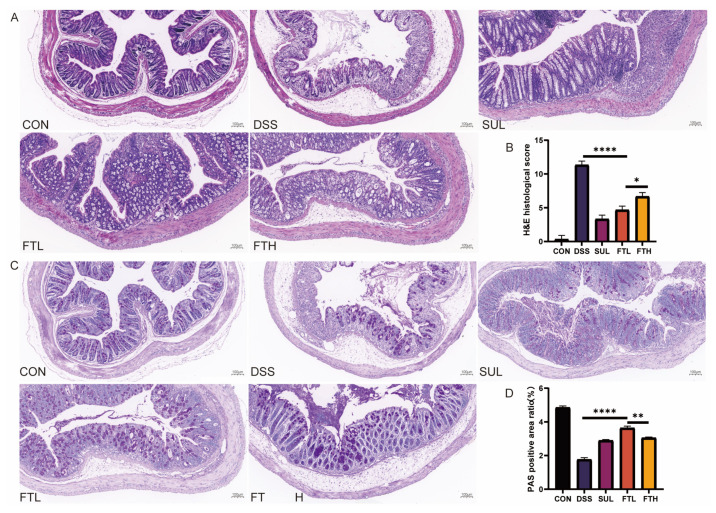
H&E and PAS staining of colon tissue of mice in each group (×10). (**A**) H&E section of mice intestinal tissue. (**B**) Histological score of H&E section. (**C**) PAS section of mice intestinal tissue. (**D**) Mucus positive rate in PAS section. *, *p* < 0.05; **, *p* < 0.01; ****, *p* < 0.0001; ns indicates that the difference is not significant.

To further explore the protective effect of FBT on the mice colon structure, we performed histomorphometric analysis to evaluate the inflammatory cell infiltration and mucosal damage in the mice colon (Figure 3). The results of H&E stained sections indicated that the colon morphology of the mice in the CON group was normal, the villi were neatly arranged, and no obvious inflammatory cell infiltration and damage were observed; the mice in the DSS group showed severe acute colitis, with disordered intestinal cell arrangement, thinning of the muscle layer, villous rupture, accompanied by a large amount of inflammatory infiltration, edema in the intestinal mucosal layer, and disappearance of the superficial epithelium in goblet cell nuclei; whereas, the pathological morphology of the FT group was relatively complete, with higher villus integrity and less inflammatory cell infiltration. The results of the tissue disease index also suggested that the FBT group was significantly lower than the DSS group (*p* < 0.0001). In particular, the protective effect of 400 mg/kg FBT extract on the colon structure and mucosa was better than that of 200 mg/kg FBT extract (*p* < 0.001).

PAS staining of colon sections revealed a significant reduction in goblet cell numbers and uneven mucin distribution in the DSS group compared to the CON group (Figure 3C,D). However, after the intervention of FBT, the number of colonic goblet cells and mucin secretion were restored, especially in the FTL group, which had higher mucin secretion in the colon of mice than the FTH group (*p* < 0.01). Therefore, FBT had the effect of protecting the structure and functional integrity of the intestine in mice with DSS-induced enteritis. The intervention with FBT increased the number of goblet cells and mucin. In summary, FBT extract could reduce the damage of DSS to the colon of mice and protect the integrity of the intestinal structure.

### 3.5. Effect of FBT on Intestinal Barrier in Colitis Mice

**Figure 4:**
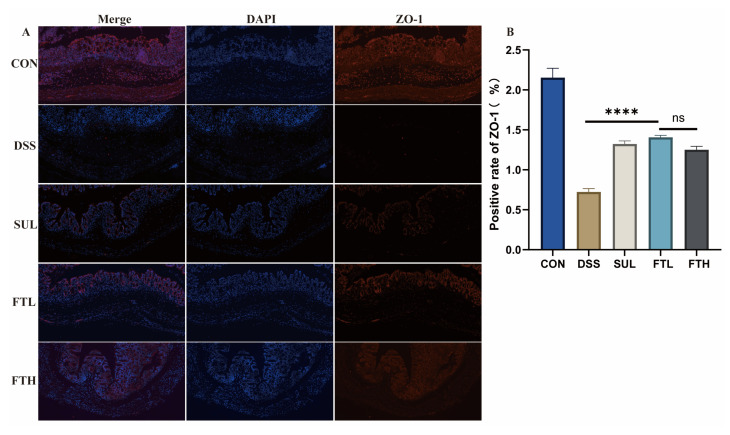
FBT protects the intestinal barrier protein ZO-1 in mice with colitis (×10). (**A**) ZO-1 protein expression in the colons. (**B**) Positive rate of ZO-1. ****, *p* < 0.0001; ns indicates that the difference is not significant.

Morphological analysis revealed that oral administration of FBT could reduce the damage of DSS to colonic goblet cells and mucosal proteins; we, therefore, speculated that FBT extract may also have a certain protective effect on intestinal barrier proteins. Thus, we used immunofluorescence technology to detect the expression of the key barrier protein ZO-1 in the colon of enteritis mice (Figure 4). The results demonstrated that ZO-1 protein expression in the colons of DSS-treated mice was significantly lower than in the CON group (*p* < 0.0001), consistent with the pathological findings from colon tissue analysis. However, after FBT intervention, the expression of ZO-1 protein was significantly increased (*p* < 0.0001) and was similar to the expression level of the CON group. The above results suggested that FBT could effectively alleviate intestinal barrier damage in mice with DSS-induced enteritis.

### 3.6. Effects of FBT on the Diversity of Gut Microbiota in Mice with Colitis

**Figure 5:**
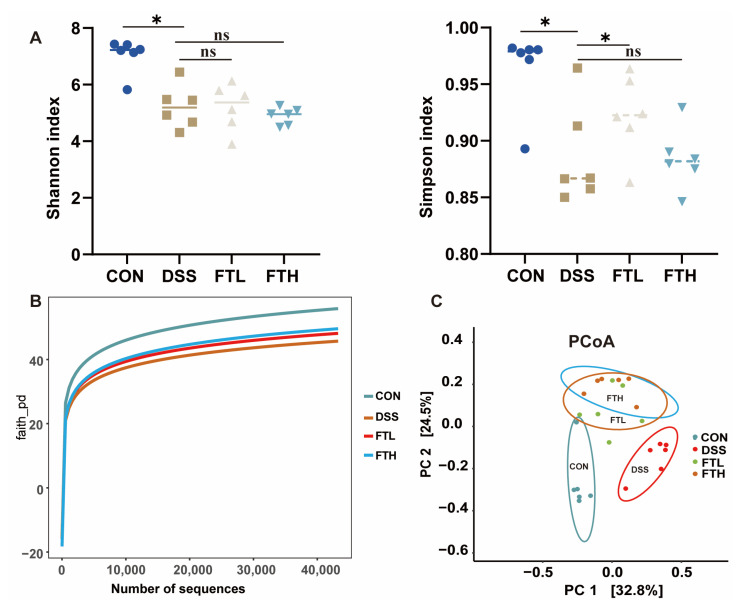
Effect of FBT on the diversity of gut microbiota in mice with DSS-induced colitis. (**A**) Simpson and Shannon index of gut microbiota. (**B**) Dilution curve of Gut microbiota. (**C**) PCoA analysis of gut microbiota. *, *p* < 0.05; ns indicates that the difference is not significant.

The intestine and its microorganisms are closely related to the host’s physiological, nutritional, and immune functions. The diversity of the intestinal ecosystem is of great significance to the metabolic balance of the host. Gut microbiota is critical to the development of colitis. Therefore, we collected cecal contents and performed PCR amplification and sequencing of the V3–V4 region of 16S rRNA. The effect of FBT on the gut microbiota composition of colitis mice was evaluated by 16S rRNA sequencing. The dilution curves tended to saturate (Figure 5B), indicating that sequencing coverage was sufficient for further data analysis. Analysis of fecal sequencing data of mice in different groups demonstrated that compared with the CON group, the Simpson index and Shannon index of the DSS group were significantly reduced (Figure 5A). After the intervention of FBT, the Simpson index and Shannon index of the FBT group increased, suggesting that FBT can restore the decrease in the diversity and richness of gut microbiota in mice with DSS-induced enteritis (Figure 5A). PCoA analysis demonstrated differences between gut microbiota compositions within the same group, and the figure indicated a unique clustering of microbial community compositions from different groups (Figure 5C). The results suggested that DSS changed the gut microbiota composition, as there were obvious differences between the DSS group and the healthy group. The FTL and FTH groups were separated from the DSS group in the PCoA analysis, indicating that FBT has a regulatory effect on the microbial community composition of mice with DSS-induced colitis.

### 3.7. Effects of FBT on Gut Microbiota in Colitis Mice

**Figure 6:**
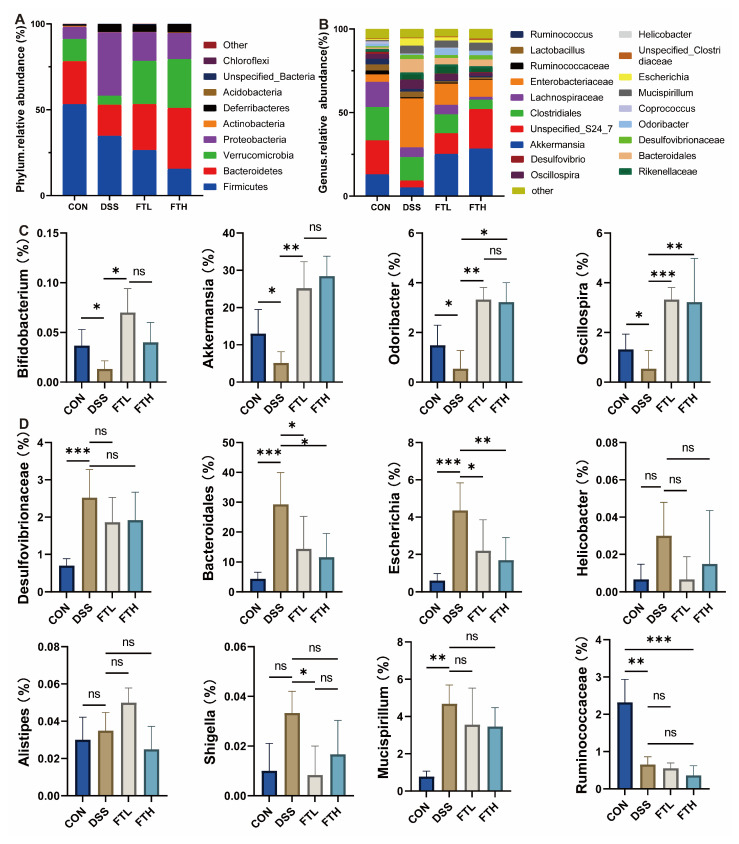
Effect of FBT on the gut microbiota of mice with DSS-induced colitis. (**A**) The bacterial flora at the phylum level. (**B**) The bacterial flora at the family level. (**C**) The beneficial bacterial flora at the genus level. (**D**) The harmful bacterial flora at the genus level. *, *p* < 0.05; **, *p* < 0.01; ***, *p* < 0.001; ns indicates that the difference is not significant.

Further analysis of the 16S rRNA sequencing results showed that 27 phyla, 67 classes, 128 orders, 216 families, 351 genera, and 429 species of bacteria were detected in all samples. At the phylum level (Figure 6A), the gut microbiota of normal mice mainly includes *Firmicutes*, *Bacteroidetes*, *Verrucomicrobia*, *Proteobacteria*, and *Deferribacteres*. However, after DSS treatment, the relative abundance of *Firmicutes*, *Proteobacteria*, and *Deferribacteres* increased, while the relative abundance of *Bacteroidetes* and *Verrucomicrobia* decreased in the intestines of mice in the DSS group, increasing the *Firmicutes*/*Bacteroidetes* (F/B) ratio. However, the intake of FBT extract helped alleviate this trend, reducing the abundance of *Firmicutes* and increasing the abundance of *Bacteroidetes* in the intestine, thereby changing the F/B ratio.

At the genus level (Figure 6C), compared with the CON group, the relative abundance of *Akkermansia*, *Odoribacter*, *Bifidobacterium*, and *Oscillospira* in the intestines of mice in the DSS group was reduced, while the relative abundance of *Rikenellaceae*, *Bacteroidales*, *Mucispirillum*, *Escherichia*, and *Helicobacter* increased (Figure 6D). Compared with the DSS group, the relative abundance of *Rikenellaceae*, *Bacteroidales*, *Mucispirillum*, *Escherichia*, and *Helicobacter* in the FTH group decreased, while the relative abundance of *Akkermansia*, *Odoribacter*, *Oscillospira*, and *Bifidobacterium* increased. Harmful bacteria, like *Desulfovibrionaceae*, *Escherichia*, *Shigella*, *Helicobacter,* etc., in the intestines of mice can produce endotoxins such as lipopolysaccharide. These endotoxins may destroy intestinal epithelial cells and damage the intestinal barrier, thereby promoting the occurrence of inflammation. The intake of Fu Brick tea could help to reduce the abundance of these harmful bacteria in the intestine, reduce the production of lipopolysaccharide and other endotoxins, and help to protect the intestinal epithelial cells and maintain the integrity of the intestinal barrier, thus reducing the occurrence of inflammation.

### 3.8. Analysis of Key Flora Abundance and SCFA Content

**Figure 7:**
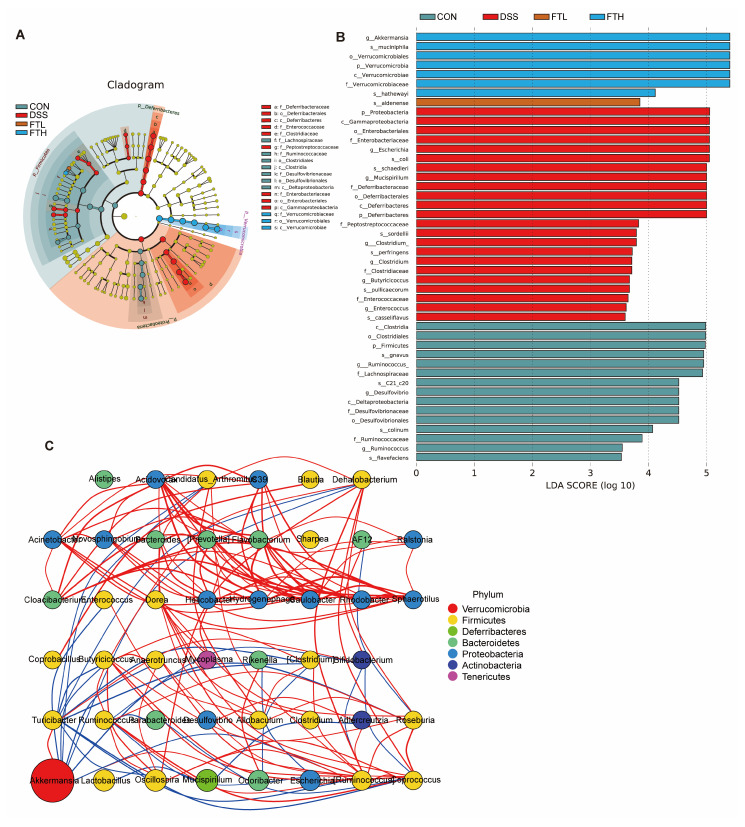
Gut microbiota LDA and Spearman correlation coefficient analysis. (**A**,**B**) Linear discriminant analysis (LDA > 2.0). (**C**) Spearman correlation coefficient analysis. Note: Comparison of the interaction network diagrams of the enriched species in each sample group. The circle represents a species, the size represents its relative abundance, different colors represent different species phylum classifications, and the lines between the circles represent the relationship between the two species. The correlation is significant (*p*-value < 0.05). The line color red represents a positive correlation, and blue represents a negative correlation. The thicker the line, the greater the absolute value of the correlation coefficient.

The size of the linear discriminant analysis (LDA) value reflects the degree of contribution of microorganisms to the differences between groups. The larger the value, the more significant the difference of this microorganism between different groups. LDA demonstrated that compared with the CON group, the main characteristic bacterial groups in the DSS group were *Escherichia*, *Clostridium*, *Mucispirillum*, *Butyricicoccus*, etc., while in the FTH group, they were *Akkermansia*, *Verrucomicrobiaceae*, etc. This showed that oral administration of FBT extract could inhibit the reproduction of *Escherichia*, *Clostridium*, *Mucispirillum*, *Butyricicoccus*, and other bacteria and promote the growth of *Akkermansia*, *Verrucomicrobiaceae*, and other bacteria (Figure 7A,B).

Spearman correlation coefficient analysis was performed based on the relative abundance between species samples to obtain the mutual relationships between species within a sample or a sample group, and then a species interaction network was constructed using visualization software (Figure 7C). This analysis showed that *Akkermansia* is an important bacterial species at the genus levels. *Akkermansia* had a positive correlation with *Oscillospira*, *Turicibacter*, etc., and a negative correlation with *Rikenella*, *Escherichia*, *Ruminococcus*, etc. This indicated that FBT might affect the structure and function of the intestinal microbiome mainly by regulating *Akkermansia* and other bacterial groups.

**Figure 8:**
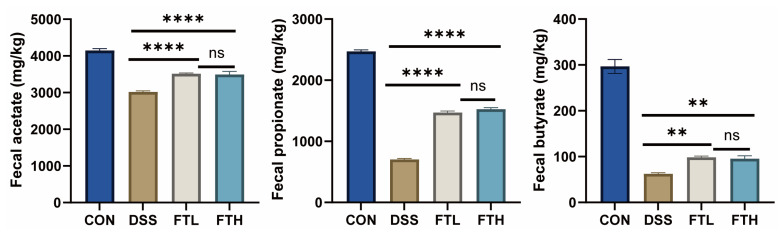
Effects of FBT on the content of SCFAs in mice. **, *p* < 0.01; ****, *p* < 0.0001; ns indicates that the difference is not significant.

Short-chain fatty acids (SCFAs) have a certain protective effect on colitis. They can promote the growth and repair of colon mucosal cells, enhance the integrity of the colon mucosal barrier, reduce the growth of harmful bacteria, inhibit inflammatory response, and regulate the balance of the immune system. To further explore the effect of FBT on SCFAs (Figure 8), we measured the fecal concentrations of acetate, propionate, and butyrate. Compared with the CON group, DSS treatment significantly reduced the contents of acetate, propionate, and butyrate. The production of acetate, propionate, and butyrate content was significantly improved after oral administration of BFT extract.

### 3.9. Correlation Analysis Between Gut Microbiota Species and Key Indicators of Colitis

**Figure 9:**
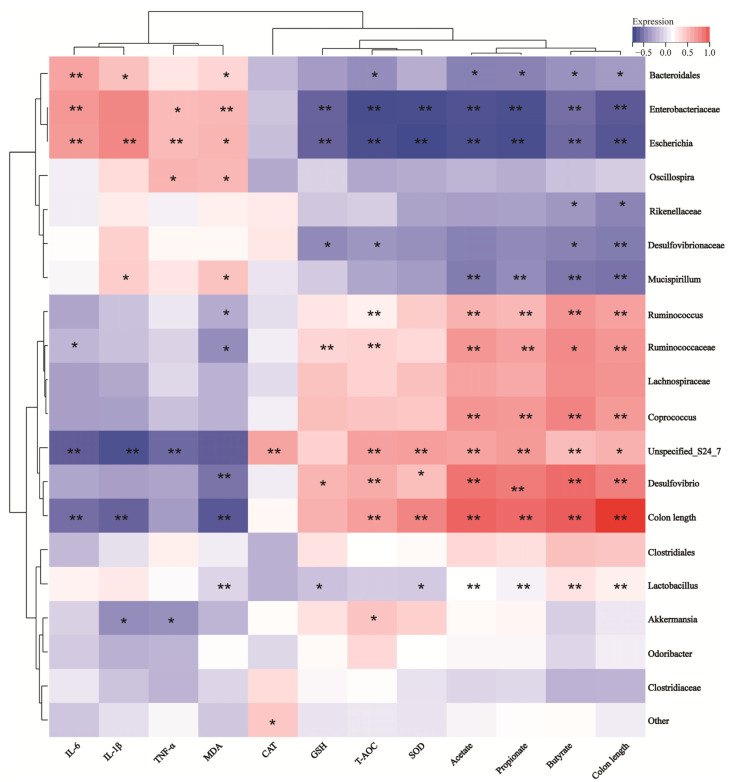
Correlation analysis between UC disease phenotype data and gut microbiota at genus. *, *p* < 0.05; **, *p* < 0.01.

To analyze whether there is a significant correlation between intestinal microbiota species and colitis phenotype data, we calculated the Spearman correlation coefficient between the inflammation phenotype data and the microbial genus levels. According to the results in Figure 9, the antioxidant index showed a consistent trend with the length of the colon, and the inflammatory factor MDA corresponded to the antioxidant index. This suggested that DSS-induced colon shortening can be alleviated by reducing intestinal inflammation and improving intestinal antioxidant properties. Further analysis of the relationship between various factors and gut microbiota demonstrated that *Akkermansia* and *Lactobacillus* were negatively correlated with TNF-α and IL-1β and positively correlated with colon length, SOD, GSH, etc. On the contrary, *Enterobacteriaceae*, *Escherichia*, *Mucispirillum*, *Desulfovibrionaceae*, etc., were positively correlated with TNF-α, IL-1β, and MDA and negatively correlated with colon length, SOD, GSH, etc. This suggested that the increase in *Enterobacteriaceae*, *Escherichia*, *Mucispirillum*, *Desulfovibrionaceae*, and other bacterial groups might aggravate intestinal inflammation, while the proliferation of *Akkermansia* and *Lactobacillus* might reduce intestinal inflammation, thereby improving colon shortening.

## 4. Discussion

IBD is a complex inflammatory condition of the colon, affecting millions of people globally each year [9]. Dextran sodium sulfate (DSS) is widely used to induce IBD in animal models. DSS disrupts the intestinal mucosal barrier, increases intestinal permeability, and allows foreign antigens to enter the lamina propria, triggering immune and inflammatory responses that lead to IBD-like pathology. It has been reported that DSS-induced mice often develop symptoms such as anorexia nervosa, weight loss, diarrhea, hematochezia, and shortened colon [22].

Oral administration of FBT extract could, to a certain extent, reduce pathological conditions such as diarrhea, blood in the stool, and weight loss in colitis model mice and had a certain protective effect on the intestinal tract of mice (Figure 1).

In the process of DSS-induced colitis, intestinal epithelial cells are damaged, and intestinal permeability is increased, leading to an imbalance of inflammatory cytokines and abnormal immune responses, ultimately triggering systemic inflammation [23]. Inflammatory cytokines, such as IL-1β, IL-6, and TNF-α, play an important role in regulating the intestinal innate immune response. Therefore, reducing pro-inflammatory factors in serum and reducing inflammatory response is an important target in the treatment of IBD. In this study, the levels of IL-1β, IL-6, and TNF-α in enteritis model mice were significantly higher than those in the control group, indicating that systemic inflammation was triggered when DSS-induced colitis. In fact, patients with colitis had elevated levels of IL-1β, IL-6, and TNF-α. After the intervention of FBT, the levels of IL-1β, IL-6, and TNF-α in the serum of enteritis model mice were reduced, and the contents of antioxidant factors such as T-AOC and SOD in the intestines were significantly increased, indicating that FBT is effective in treating colitis via anti-inflammatory and antioxidant influences (Figure 2). The potential direct effects of FBT’s bioactive compounds, such as polyphenols, polysaccharides, and other phytochemicals, may be related to reducing inflammation, protecting the intestinal barrier, and modulating immune responses. These compounds may exert direct antioxidant and anti-inflammatory effects independent of gut microbiota modulation.

Modern medicine also emphasizes that the destruction of intestinal mucosal barrier integrity is the pathological basis for the occurrence and development of various metabolic diseases, intestinal diseases, and various infectious diseases [24]. Research shows that when the body is stimulated by stress factors such as pathogen invasion, extensive use of antibiotics, irregular diet, and emotional abnormalities, it usually leads to damage of the intestinal mucosal barrier and increases intestinal permeability. This allows harmful bacteria and their metabolites, such as endotoxin (LPS), to pass through the damaged intestinal barrier, enter the bloodstream, and then move throughout the body, causing chronic inflammatory reactions in multiple tissues and organs of the body. This increases the risk of obesity, diabetes, etc. Metabolic diseases and susceptibility to multiple organ infectious diseases [25]. The intestinal mucosal barrier is mainly composed of three parts: the physical barrier, composed of the epithelial cell layer and the mucosal layer; the immune barrier, composed of immune cells in the intestine and antibodies secreted by the intestinal mucosa; and the microbial barrier, composed of intestinal commensal flora [26]. These three work together to maintain the integrity of the intestinal barrier. Therefore, improving the integrity of the physical barrier, immune barrier, and microbial barrier of the intestinal mucosa helps to maintain the function of the intestinal mucosal barrier, which is a key goal in maintaining the body’s healthy state.

Damage to the intestinal epithelial barrier, one of the symptoms of IBD, not only leads to inflammation but also further leads to bacterial translocation and the entry of other antigens. At the same time, according to the results of pathological sections of the mice colon, DSS-induced colitis resulted in serious pathological damage to the colon, including muscle layer thinning, villus disruption, obvious inflammatory infiltration, mucosal edema, reduction in the number of goblet cells, and mucosal infiltration, decreasing protein secretion [27]. Defective intestinal mucus production and reduced goblet cell numbers are associated with the development of IBD. Therefore, maintaining the integrity and tightness of the intestinal barrier is also a key goal in treating IBD. Tight junction proteins in the intestine are related to the impairment of intestinal epithelial barrier function. ZO-1 is a major protein in tight junctions and is closely related to epithelial integrity. It is often used as a marker of the intestinal mechanical barrier [28]. The protein expression of ZO-1 in the colon was detected by immunofluorescence, and it was found that oral administration of FBT can restore the intestinal barrier damage in the mice colon caused by DSS, reduce inflammatory cell infiltration, and protect the functional morphology of the intestine (Figure 4).

Studies have found that the number of microorganisms in the human intestine is approximately 10^14^, which is approximately 10 times the number of cells in the human body, including commensal bacteria, probiotics, and pathogenic bacteria. They play important roles in nutrient absorption, host defense, immune development, etc. [29,30]. The balance of gut microbiota is closely related to multiple physiological functions of the host, such as immunity, nutritional metabolism, and overall health [31,32]. When the ecological balance of the gut microbiota is disrupted by invasive antigens, immune system activation, and inflammatory factor production, this may lead to the occurrence and progression of a variety of diseases [33,34]. Disturbance of gut microbiota has been considered a key factor in the pathogenesis of IBD. Studies have shown that in mice with DSS-induced colitis, the diversity and stability of the gut microbiota are significantly reduced, resulting in a chaotic state of the gut microbiota. In addition, gut microbiota adheres closely to intestinal epithelial cells, forming a natural and unique biological mucosal barrier, which can effectively prevent the invasion of foreign pathogens [35].

By detecting the changes in intestinal microorganisms, we can perform microbial diversity analysis. Before conducting microbial diversity analysis, it is necessary to randomly select a certain number of sample sequences from the sample and count the number of species they represent to construct a dilution curve. This helps to verify whether the amount of sequencing data is sufficient to fully reflect the species diversity in the sample, thereby indirectly reflecting the abundance of each species in the sample [36]. To a certain extent, as the number of sequencing increases, when the curve levels off, it indicates that the number of species in the environment will not increase significantly, and the number of sequences in the sample is sufficient for subsequent analysis of intestinal microbial diversity. In community ecology, species diversity is usually evaluated through alpha diversity analysis, which mainly includes species richness and evenness [37]. It could demonstrate the effect of FBT on the species diversity of intestinal microorganisms in mice with enteritis (Figure 6). DSS caused a decrease in the richness and diversity of intestinal microorganisms in mice. Oral administration of FBT extract could alleviate this trend to a certain extent and increase the diversity and richness of intestinal microbial species. However, in this study, there were certain limitations in the ability of FBT extract to restore the diversity of gut microbiota. This might be due to the high concentration of DSS reaching 3%, which caused irreversible damage to the gut microbiota.

*Akkermansia* is a strictly anaerobic bacterium that uses intestinal mucin as its sole carbon and nitrogen source to grow. The metabolic activities of *Akkermansia muciniphila* (e.g., mucin degradation, SCFA production, and immune modulation) mainly exist in the intestinal mucosa layer and play an important role in maintaining intestinal barrier function [38,39]. *Akkermansia muciniphila* is known to degrade mucin and promote the regeneration of the mucus layer, which plays a critical role in maintaining intestinal barrier integrity and preventing pathogen invasion [40,41]. At the same time, *Akkermansia* can also produce SCFAs, such as acetate, propionate, and butyrate, to provide the necessary nutrients for the intestines, maintain gut homeostasis, reduce inflammation, and support epithelial cell health [42]. *Akkermansia* interacts with other gut microbiota and influences the production of SCFAs, which play an important role in modulating immune responses, including its anti-inflammatory effects and its ability to promote regulatory T-cell differentiation, which contributes to the gut–immune balance. Some studies have found that the administration of *Akkermansia* can effectively alleviate colon inflammation in mice with colitis [43]. In this study, FBT extract not only increased the diversity of gut microbiota in mice with enteritis but also helped restore the abundance of *Firmicutes* and *Bacteroidetes* in mice with DSS-induced colitis. At the genus levels, FBT extract also significantly increased the relative abundance of beneficial bacteria *Akkermansia* and *Bacteroides* in colitis mice. The effects of FBT are mediated through its prebiotic-like modulation of the gut microbiota rather than direct probiotic activity. This will include a more detailed explanation of how FBT’s bioactive compounds (e.g., polyphenols, polysaccharides) promote the growth of beneficial bacteria and inhibit harmful bacteria, thereby restoring gut homeostasis. FBT’s polyphenols and polysaccharides can serve as substrates for beneficial bacteria like *Akkermansia*, promoting their growth and enhancing their metabolic activities, such as SCFA production. These microbiota-mediated effects could further contribute to gut homeostasis and inflammation reduction. In mice with colitis, harmful bacteria such as *Desulfovibrionaceae*, *Escherichia*, *Shigella*, and *Helicobacter* are often enriched. These bacteria can produce endotoxins such as lipopolysaccharide to destroy intestinal epithelial cells, damage the intestinal barrier, and thus promote the occurrence of inflammation [44]. The intervention of FBT extract can effectively inhibit the growth of these harmful bacteria, especially the proliferation of *Desulfovibrionaceae*, *Escherichia*, and *Shigella* (Figure 7).

There is a certain relationship between SCFAs and colitis. SCFAs are metabolites produced by the fermentation of dietary fiber by probiotics in the gut, mainly including acetic acid, propionic acid, and butyric acid. These SCFAs have a variety of physiological functions in the colon, including providing energy, maintaining colon mucosal barrier function, and regulating immune response [45]. SCFAs (e.g., acetate, propionate, and butyrate) play a crucial key in maintaining gut barrier integrity, reducing inflammation, and regulating immune responses [46]. SCFAs are known to regulate immune responses by binding to G-protein-coupled receptors (GPCRs), such as GPR41 and GPR43, on immune cells [47]. This interaction inhibits the activation of pro-inflammatory pathways, including NF-κB, and promotes the differentiation of regulatory T cells (Tregs), which are crucial for maintaining intestinal immune homeostasis [48]. SCFAs have been demonstrated to help repair damaged epithelial cells. Studies have shown that SCFAs can increase the thickness of intestinal mucosa, promote the proliferation and differentiation of intestinal epithelial cells, promote the synthesis of tight junction protein of intestinal epithelial cells, inhibit intestinal permeability, and thus enhance the mechanical barrier function of intestinal mucosa [49]. Butyrate, in particular, serves as a primary energy source for colonocytes, enhancing epithelial barrier integrity by upregulating tight junction proteins (e.g., ZO-1, occludin) and promoting mucus production [50,51]. This helps prevent bacterial translocation and reduces inflammation. SCFAs can also modulate gene expression through histone deacetylase (HDAC) inhibition, leading to the upregulation of anti-inflammatory genes and downregulation of pro-inflammatory cytokines. SCFAs can also nourish immune cells, promote the secretion of mucin, lubricate the intestine, and reduce the adhesion of pathogenic bacteria on the intestinal mucosa [52]. To further explore the effects of oral FBT on SCFA production, we measured fecal concentrations of acetate, propionate, and butyrate (Figure 8). DSS significantly reduced all three SCFAs, but oral FBT significantly improved propionate and butyrate production.

While previous studies have explored the anti-inflammatory and antioxidant effects of Fu Brick tea (FBT), our study provides a more comprehensive evaluation by integrating gut microbiota modulation, intestinal barrier protection, and SCFA production. We demonstrated that FBT aqueous extract promotes the growth of beneficial bacteria (e.g., *Akkermansia*), inhibits harmful bacteria (e.g., *Desulfovibrioceae*, *Escherichia*, *Helicobacter*), and restores intestinal homeostasis in a dose-dependent manner. Additionally, our correlation analysis revealed significant relationships between specific microbial taxa and colitis phenotypes, providing new insights into the mechanisms underlying FBT’s protective effects. These findings contribute to the growing body of evidence supporting the use of FBT as a dietary supplement for colitis management.

However, it is important to note that our study shows a significant association between FBT consumption and changes in gut microbiota composition but does not establish direct causality. Further mechanistic studies, such as fecal microbiota transplantation (FMT) experiments, are needed to confirm whether the observed microbial changes are directly responsible for FBT’s protective effects on colitis. Future research should also explore the dose-dependent effects of FBT to determine the optimal therapeutic dose for colitis management. Additionally, isolating and evaluating individual bioactive components (e.g., polyphenols, polysaccharides) will help identify the specific compounds driving the observed anti-colitis effects. Studies using germ-free animal models or in vitro experiments could further delineate the direct and indirect mechanisms of FBT’s active compounds, independent of gut microbiota mediation.

## 5. Conclusions

FBT extract significantly improved the disease index in colitis mice, reduced inflammatory responses, protected the intestinal barrier protein (ZO-1), and maintained intestinal structural integrity. Additionally, FBT intake increased gut microbiota diversity, promoted the growth of beneficial bacteria (*Akkermansia*), inhibited the proliferation of harmful bacteria (*Desulfovibrioceae*, *Escherichia*, and *Helicobacter*), restored intestinal homeostasis, and alleviated colitis symptoms, including diarrhea. FBT can be used as a dietary supplement to reduce intestinal inflammatory response, maintain the balance of gut microbiota and gut microbiota, and protect intestinal health. However, it is important to note that these findings are preliminary and based on an animal model. Further studies, including clinical trials in humans, are needed to confirm the efficacy, safety, and mechanisms of FBT in managing colitis and promoting intestinal health.

## Figures and Tables

**Table 1:** Scoring criteria of disease activity index.

Stool	Bleeding	Weight Loss (%)	Score
Normal	Normal	Unchanged	0
-	-	1–5	1
Loose	Occult blood positive	5–10	2
-	-	10–15	3
Diarrhea	Overt bleeding	>15	4

## Data Availability

The original contributions presented in this study are included in the article. Further inquiries can be directed to the corresponding authors.

## Abbreviations

The following abbreviations are used in this manuscript:

FBT	Fu Brick tea
ZO-1	Intestinal barrier protein
IBD	Inflammatory bowel disease
UC	Ulcerative colitis
CD	Crohn’s disease
ROS	Reactive oxygen species
IL-1β	Interleukin-1 beta
IL-6	Interleukin-6
TNF-α	Tumor necrosis factor-α
SOD	Superoxide dismutase
CAT	Catalase
GSH	Glutathione
MDA	Malondialdehyde
T-AOC	Total Antioxidant Capacity
DSS	Dextran sodium sulfate
CON	Normal control group
DSS	DSS enteritis model group
SUL	Positive control group (treated with sulfasalazine)
FTL	200 mg/kg FBT group
FTH	400 mg/kg FBT group
LDA	Linear discriminant analysis
F/B	The ratio of Firmicutes/Bacteroidetes
SCFAs	Short-chain fatty acids
HPLC	High-performance liquid chromatography
H&E	Hematoxylin and eosin staining
PAS	Periodic acid-Schiff staining
FID	Flame ionization detector
ANOVA	One-way analysis of variance
